# The effects of Chinese botanical lotion and pimecrolimus cream in the treatment of chronic perianal eczema: a randomized clinical trial

**DOI:** 10.3389/fphar.2025.1673112

**Published:** 2025-11-12

**Authors:** Lixia Lai, Yanmei Wang, Luping Wen, Shaozhao Wang, Si Chen, Chao Gu, Zeye Zhang

**Affiliations:** 1 Department of Proctology, China-Japan Friendship Hospital, Beijing, China; 2 Department of Coloproctology, The Affiliated Hospital of Xuzhou Medical University, Xuzhou, China; 3 Department of Anorectal Word, Jinan Central Hospital, Jinan, China; 4 School of Life Sciences, Beijing University of Chinese Medicine, Beijing, China; 5 Department of Anorectal Surgery, Affiliated Hospital of Shandong University of Traditional Chinese Medicine, Jinan, China; 6 School of Basic Medical Sciences, Zhejiang Chinese Medical University, Hangzhou, China

**Keywords:** chronic perianal eczema, pimecrolimus cream, Chinese botanical lotion, fumigation and sitz-bath therapy, anal pruritus lotion

## Abstract

**Background:**

Fumigation and sitz-bath therapy are traditional Chinese medicine (TCM) practices that offer unique benefits for managing chronic perianal eczema (CPE). This study aimed to investigate the clinical efficacy of the traditional Chinese herbal lotion (anal pruritus lotion, APL) combined with pimecrolimus cream in CPE treatment.

**Methods:**

Patients with CPE admitted between October 2019 and March 2022 were analyzed. The control group was given basic therapy with pimecrolimus cream, whereas the treatment group received pimecrolimus cream combined with APL under fumigation and sitz-bath therapy. We recorded and compared baseline patient information and clinical symptoms pre- and post-therapy, including clinical symptom scores, the eczema area and severity index (EASI), pruritus visual analogue scale (VAS) scores, dermatology life quality index (DLQI), and efficacy evaluations. Additionally, safety assessments and follow-up surveys were performed, as well.

**Results:**

Baseline data were comparable between the treatment and control groups. Post-therapy, the treatment group exhibited significantly improved outcomes compared to the control group in eczema severity, pruritus VAS scores, and dermatology life quality, with lower relapse rates (*P* < 0.05). Safety evaluations suggested that the treatments were safe and reliable.

**Conclusion:**

The combination of pimecrolimus cream and APL is more effective in the treatment of CPE than pimecrolimus cream alone, providing a promising new approach for the clinical management of perianal eczema.

**Clinical Trial registration:**

International Traditional Medicine Clinical Trial Registry, http://itmctr.ccebtcm.org.cn/en-US/Home/ProjectView?pid=58f1a168-bdf1-4e2a-a9bd-cf0f4a3ec06f as ITMCTR 2024000576.

## 1 Introduction

Chronic perianal eczema (CPE) is a recurrent inflammatory skin condition that primarily affects the perianal region, often extending to the perineum and external genitalia (Kränke et al., 2006). Clinically, CPE presents as erythema, scaling, and infiltrative hypertrophy, accompanied by persistent pruritus that typically intensifies at night (Fang et al., 2022). The chronic nature and high recurrence rate of CPE significantly impact patients’ daily functioning, sleep, and social interactions, potentially leading to psychological distress such as anxiety, depression, and stress-related disorders (Sailer et al., 1998; Weyandt et al., 2020). Given its complex pathogenesis and the persistent disruption of quality of life, CPE poses considerable challenges for both treatment and prevention of relapses (Schauber et al., 2009; Gola et al., 2017).

Conventional therapies, including glucocorticoids, antihistamines, and immunosuppressants, provide symptom relief but are usually associated with notable adverse effects such as perianal skin atrophy, keratin thickening, immune dysfunction, and high relapse rates following treatment withdrawal (Weyandt et al., 2020; Rattner, 1955; Wollenberg et al., 2018; Berger et al., 2006). In light of these limitations, there is an urgent clinical need for more effective and safer treatment alternatives.

Fumigation and sitz-bath therapy, ancient practices in traditional Chinese medicine (TCM), have demonstrated unique advantages in treating CPE. Modern pharmacological studies indicate that TCM fumigation combines thermal effects with medicinal properties, thereby enhancing local drug penetration and promoting dual therapeutic effects (Wang et al., 2021; Zhang et al., 2018). When followed by sitz-bath therapy, this treatment delivers medication directly to the affected areas, offering a safe and practical approach that may reduce recurrence without significant side effects (Kang et al., 2021). Based on a substantial body of clinical research, we propose a traditional Chinese medicine formula (anal pruritus lotion, APL) that has the effect of “wind-damp-heat-stasis” (He et al., 2024).

Pimecrolimus cream, a selective topical calcineurin inhibitor, exerts anti-inflammatory effects by suppressing T-cell activation and reducing the production of proinflammatory cytokines such as IL-2, IL-4, and TNF-α. In addition, it modulates neuroimmune interactions by blocking the release of neuropeptides closely associated with pruritus in chronic eczema, such as substance P (Cui et al., 2021; Hua et al., 2022). Furthermore, APL, comprised of traditional Chinese botanical drugs known for their anti-inflammatory, antipruritic, antibacterial, and tissue-regenerating properties, can enhance the overall therapeutic response. In particular, the fumigation and sitz-bath process increases local blood flow and facilitates drug absorption through the skin’s sweat glands and follicles, thereby synergistically boosting the transdermal effects of pimecrolimus.

In this study, we investigated the combined use of APL and pimecrolimus cream to enhance treatment efficacy and reduce the relapse activity in CPE. This prospective, multicenter, randomized controlled trial was designed to evaluate the therapeutic effect and safety of APL in combination with pimecrolimus. The treatment group received APL along with pimecrolimus cream, while the control group received pimecrolimus cream alone. Clinical symptoms, EASI scores, pruritus VAS scores, overall efficacy, and skin-related quality of life were comprehensively assessed to provide robust clinical evidence supporting the integration of APL in the treatment of CPE.

## 2 Methods

### 2.1 Ethical statement

This study was conducted at the China-Japan Friendship Hospital. Participants were included after providing informed consent, in accordance with the general guidelines of the Declaration of Helsinki. The study received approval from the Clinical Research Ethics Committee of China-Japan Friendship Hospital (no. 2020-83-K49) and was registered with the International Traditional Medicine Clinical Trial Registry (ITMCTR 2024000576).

### 2.2 Subject recruitment and grouping

**FIGURE 1:**
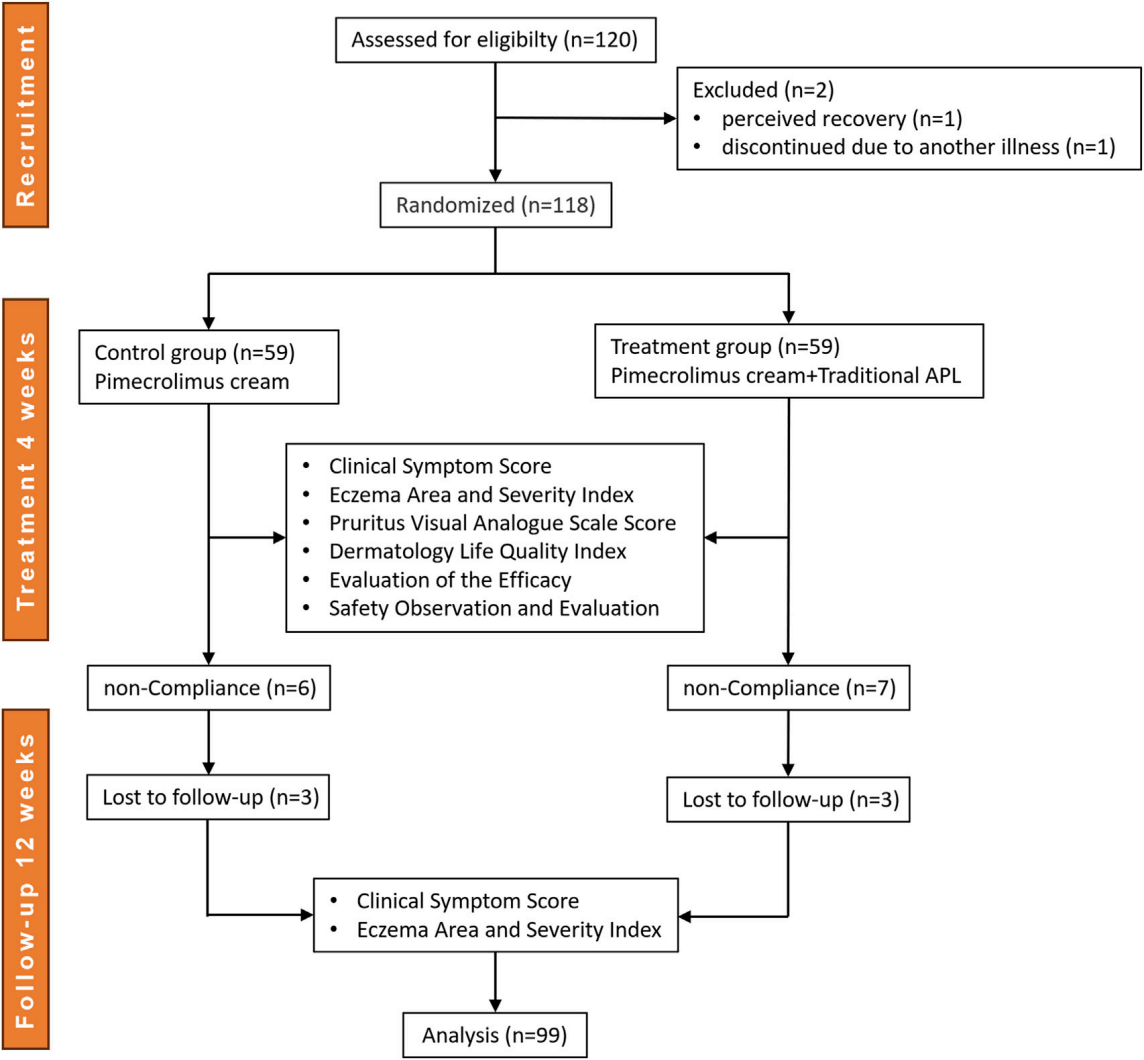
Flow diagram.

This randomized, controlled trial was conducted between October 2019 and March 2022, during which a total of 120 patients with perianal eczema were recruited from the China-Japan Friendship Hospital (Beijing, China), the Affiliated Hospital of Xuzhou Medical University (Jiangsu, China), and Jinan Central Hospital of Shandong Province (Shandong, China). Random sequences were generated using SPSS 22.0 software based on the random number table method. Eligible participants were assigned to either the treatment group (APL with pimecrolimus cream) or the control group (pimecrolimus cream alone) in a 1:1 allocation ratio. Of the initial participants, 21 patients dropped out of the study, resulting in 99 patients who completed the trial. Data from the treatment group (n = 49) and the control group (n = 50) were included in the final analysis. The enrollment process is outlined in Figure 1.

The diagnostic criteria were as follows: 1) the disease confined to the perianal area, featuring skin thickening, infiltration, pigmentation or hypopigmentation, rough surface, crust-like scales, individual mossy changes of different degrees, and clear edges; and 2) obvious pruritus that is paroxysmal and aggravated at night.

The inclusion criteria were as follows: 1) a diagnosis of chronic eczema with lesions confined to the perianal area; 2) age between 18 and 65 years; 3) no use of anti-inflammatory, antihistamine, or immunosuppressant medications within 2 weeks prior to treatment, and no local application of ointment-type medications to the anal region; 4) voluntary acceptance of the physician’s diagnosis and treatment; and 5) signing of the informed consent form.

The exclusion criteria were as follows: 1) age under 18 or over 65 years; 2) presence of other perianal diseases such as anal fissure, mixed hemorrhoids, anal fistula, or perianal abscess; 3) perianal eczema stemming from intestinal inflammatory diseases, parasites, contact dermatitis, or other causes; and 4) concomitant conditions affecting vital organs (e.g., heart, liver, kidney), malnutrition, immune system diseases, or related drug allergies.

The shedding criteria were as follows: 1) failure to meet inclusion criteria or incomplete information; 2) non-compliance with prescribed medications after inclusion; 3) voluntary withdrawal from the clinical trial; and 4) occurrence of serious adverse events (e.g., complications, significant allergic reactions, comorbidities, and specific physiologic changes; Table 1).

**TABLE 1:** Enrollment process of patients.

Item	Case (n)	
Recruitment	120	
Excluded	2	
Randomized	treatment	control
	59	59
non-Compliance	7	6
Withdrawal	3	3
Serious adverse events	0	0
Analyzed	49	50

### 2.3 Treatment

Pimecrolimus cream was smeared in the perianal region of patients in both groups. The overall treatment regimen consisted of a 4-week course, with applications twice daily. In addition, the patients in the treatment group received fumigation and sitz-bath using a traditional Chinese medicine formula (anal pruritus lotion, APL) over the course of 4 weeks. The APL was composed of botanical materials: Kushen (*Sophora flavescens* Aiton) 30 g, Huangbo (*Phellodendron chinense* C.K. Schneid.) 45 g, Machixian (*Halimione portulacoides* (L.) Aellen) 45 g, Jingjie (*Schizonepeta tenuifolia* (Benth.) Briq.) 45 g, Fuping (*Lemna polyrrhiza* L.) 20 g, Chantui (*Astragalus cicadae* M.E. Jones) 15 g, Fangfeng (*Saposhnikovia divaricata* (Turcz.) Schischk.) 10 g, Difuzi (*Kochia scoparia* (L.) Schrad.), Danggui (*Angelica acutiloba* (Siebold & Zucc.) Kitag) 30 g, and Wushaoshe (*Zaocys dhumnades* (Cantor)) 15 g (He et al., 2024). The medication was supplied and validated by the China-Japan Friendship Hospital, with authentication conducted by Professor Yanmei Wang. The botanical preparations were dispensed and decocted by licensed pharmacists according to standard procedures. Each formula was decocted into approximately 400 mL of liquid. This fumigation and sitz-bath procedure was performed under the supervision of certified physicians. Patients utilized 200 mL of the solution each time, combining it with an additional 200 mL of water at a temperature of 60 °C prior to application. The fumigation process lasted 5 min, followed by a sitz-bath of 15 min in water maintained at a temperature of 37 °C–40 °C. This fumigation and sitz-bath treatment was administered twice daily.

### 2.4 Observation criteria

#### 2.4.1 Clinical symptom score

The clinical manifestations were divided into nine categories: erythema (E), oedema/infiltration/papulation (I), blister (B), exudation/escharosis (Ex), ischemia (L), chromatosis (C), scale (S), scratch (Sc), and pruritus (P). The severity of each clinical feature was scored according to the Six Area, Six Sign Atopic Dermatitis (SASSAD) scale (ranging from 0 to 3) (Rodriguez-et al., 2012). The clinical symptom scores were calculated using the formula: symptom scores = E + I + B + Ex + L + C + S + Sc + P.

#### 2.4.2 Eczema area and severity index (EASI)

According to China’s new nine-point method, the eczema area, limited to the perianal region, was estimated based on the size of the patient’s palm, with one palm equating to 1 point. The calculation standards for eczema area are as follows: areas less than or equal to one palm receive 1 point; areas covering 1-2 palms receive 2 points; 2-3 palms, 3 points; 3-4 palms, 4 points; 4-5 palms, 5 points; and areas covering 5-6 palms, 6 points (Ständer et al., 2007; Charman et al., 2002). The EASI scores were calculated using the formula: EASI = symptom score * area * 0.3.

#### 2.4.3 Pruritus visual analogue scale (VAS) score

The degree of pruritus was assessed using the VAS scores (maximum score: 10) (Hanifin et al., 2001). The evaluation methods were as follows: a 10 cm horizontal line was drawn on paper, with one end marked as 0 (indicating no itching) and the opposite end marked as 10 (indicating unbearable itch), with gradations of itching indicated along the line. Patients were instructed to indicate their levels of itching on the line.

#### 2.4.4 Dermatology life quality index (DLQI)

The DLQI was used for evaluation, consisting of 10 questions (Ucak et al., 2013). Each question was graded on a 4-point scale (0 = none, 1 = a little, 2 = a lot, 3 = very much). The total scores, adding the points of the 10 questions, ranged from 0 (indicating no impact) to 30 (indicating maximum impact).

#### 2.4.5 Evaluation of efficacy

In this study, the effect was determined by the changes in the EASI scores, calculated as the improvement rate of clinical symptom scores using the formula: improvement rate = [(EASI before treatment - EASI after treatment)/EASI before treatment] ×100%. The efficacy was categorized into 4 grades: Cure (symptom score reduced ≥90%), Significantly effective (90%>symptom score reduced ≥75%), Effective (75%>symptom score reduced ≥50%), and Ineffective (symptom score reduced <50%).

Total significant efficacy was calculated to comprehensively evaluate the overall therapeutic effectiveness of treatment. It represents the proportion of patients who achieved either complete recovery or significant improvement post-therapy. The calculation formula is as follows: Total significant efficacy (%) = (number of clinically cured patients + number of significantly effective patients)/total number of effective patients *100%.

#### 2.4.6 Safety observation and evaluation

The safety of patients during drug administration was rigorously evaluated and monitored in this study. Safety was classified as follows: Grade Ⅰ: Safe, no adverse reactions; Grade Ⅱ: Relatively safe, with mild and manageable adverse reactions allowing continued treatment; Grade Ⅲ: Safety concerns, with moderate adverse reactions that require withdrawal of the drug and special treatment; Grade Ⅳ: Suspension of the test due to severe adverse reactions, requiring immediate withdrawal or emergency treatment.

### 2.5 Follow-up survey

The treatment course lasted for 4 weeks, and a follow-up visit occurred 3 months (12 weeks) after treatment completion. Clinical symptoms and perianal eczema were recorded during the follow-up. The EASI scores were recalculated and compared to the scores obtained immediately after the treatment course.

### 2.6 Statistical methods

Statistical analyses were performed using SPSS 22.0 software. For categorical variables, the chi-square test (χ^2^ test) was employed. For continuous variables, independent t-tests were used to assess the differences, assuming equal variances between groups. Paired sample t-tests were conducted to evaluate the differences between groups before and after treatment. Pearson’s correlation coefficient was utilized to assess correlations among the measured variables prior to analysis. The sample size was calculated using the formula: N = $\frac{{\left[Z_{\alpha }\sqrt{2\bar{P}\left(1-\bar{P}\right)}+Z_{\beta }\sqrt{P_{1}\left(1-P_{1}\right)+P_{2}\left(1-P_{2}\right)}\right]}^{2}}{{\left(P_{1}-P_{2}\right)}^{2}}$ . where P_1_ and P_2_ represent the expected total efficacy rates of the treatment and control groups, respectively, and P is the average efficacy rate (Liu and Wang, 2016). Based on previous literature, the anticipated overall efficacy was estimated as 93% for the treatment group and 72% for the control group. With α = 0.05 and β = 0.10, the calculated sample size was determined to be 48 participants per group. To accommodate an expected dropout rate of 20%, 60 cases were planned for each group (1:1 allocation), resulting in a total of 120 enrolled patients. After accounting for 21 dropouts, 99 patients completed the clinical study (49 in the treatment group and 50 in the control group). A *p*-value <0.05 was considered statistically significant.

## 3 Results

### 3.1 Baseline information comparison between the two groups

A total of 99 patients who met the inclusion criteria and completed the study were analyzed, consisting of 49 in the treatment group (APL with pimecrolimus cream) and 50 in the control group (pimecrolimus cream alone). No significant differences were observed between the two groups in sex distribution, age, or disease duration (*P* > 0.05). Baseline clinical indices, including symptom scores, eczema area and severity index (EASI), pruritus visual analogue scale (VAS), and dermatology life quality index (DLQI), did not reveal significant differences between groups before treatment (*P* > 0.05). All baseline characteristics are comparable for both groups of patients with chronic perianal eczema (CPE), and detailed information is shown in Table 2.

**TABLE 2:** Baseline characteristics of patients in the treatment and control groups.

	Treatment group		Control group		*P* value
Case (n)	49		50		
	Male	Female	Male	Female	
Gender	17 (34.7%)	32 (65.3%)	22 (44.0%)	28 (56.0%)	0.343
Age (year)	41.74 ± 12.25		38.18 ± 10.27		0.121
Course of disease (month)	6.82 ± 7.47		6.84 ± 7.83		0.988
Clinical symptom score	7.29 ± 2.53		6.78 ± 2.02		0.275
EASI	4.15 ± 2.13		3.96 ± 2.01		0.647
Pruritus VAS score	5.06 ± 1.96		5.06 ± 1.96		0.998
DLQI	8.82 ± 5.12		7.92 ± 5.52		0.404

### 3.2 The effects of pimecrolimus cream with APL on overall efficacy in patients with CPE

**FIGURE 2:**
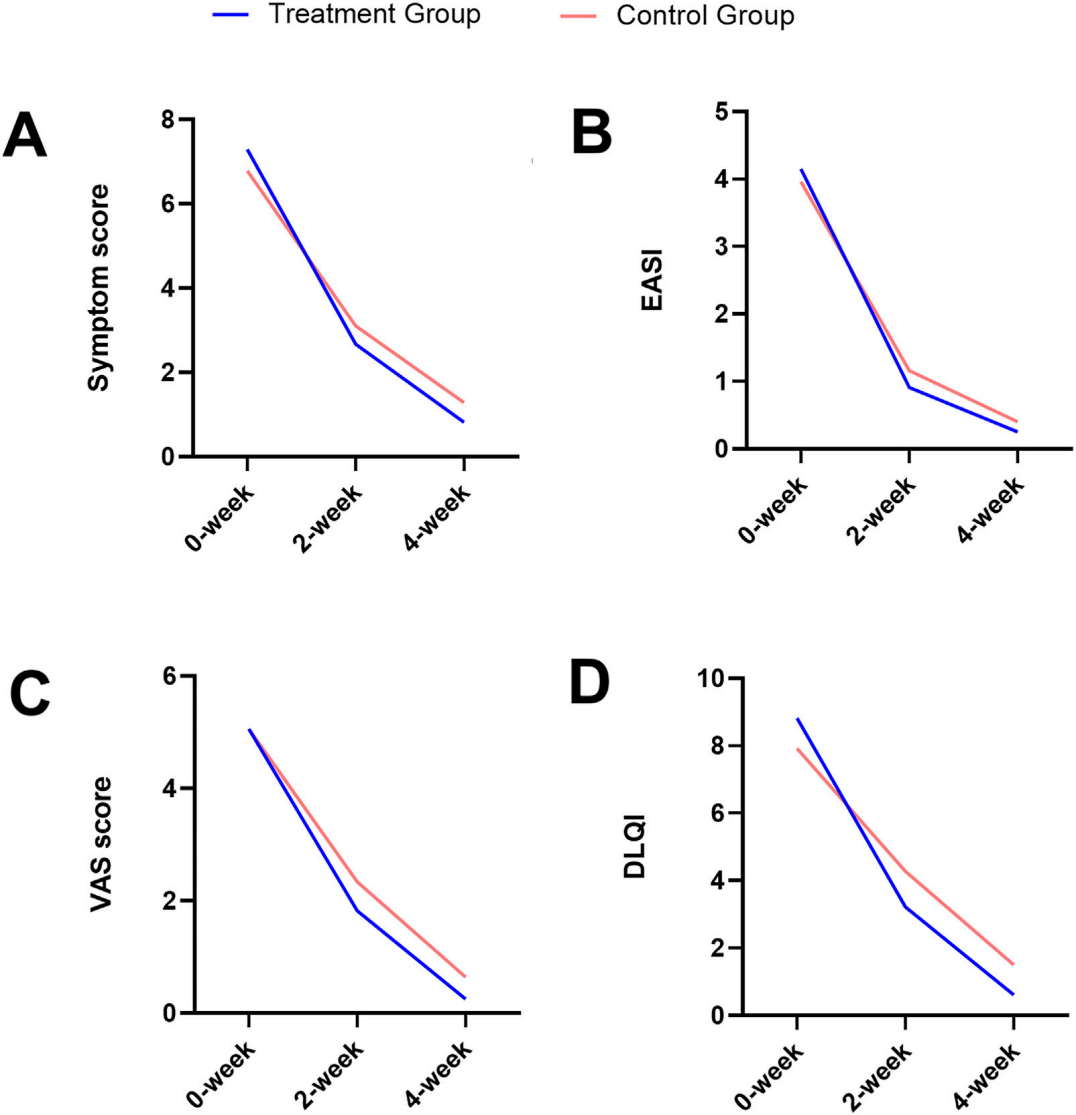
Trends over time in the clinical symptom score **(A)**, EASI **(B)**, pruritus VAS score **(C)**, and DLQI **(D)** for both the treatment and the control groups.

During a 4-week treatment course, various clinical indicators in CPE patients were meticulously analyzed for both groups, including the clinical symptom scores (Figure 2A), EASI (Figure 2B), pruritus VAS scores (Figure 2C), and DLQI (Figure 2D). Overall, all indicators exhibited a consistent downward trend over time in both groups.

**FIGURE 3:**
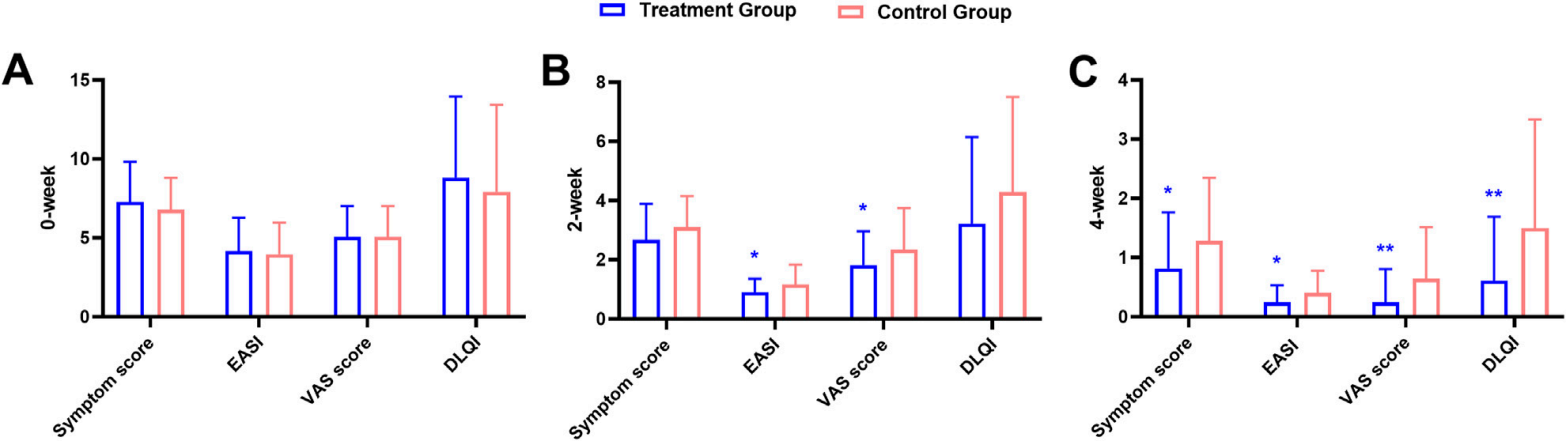
Comparison of clinical indicators at baseline **(A)**, 2-week **(B)**, and 4-week **(C)** between the treatment and the control groups during the treatment course.

At baseline, there were no significant differences between the two groups for any of these indicators (Figure 3A; Table 2). After 2 weeks of treatment, all four indicators decreased, among which the EASI (treatment group 0.91 ± 0.45 vs. control group 1.16 ± 0.67, *P* = 0.028) and the pruritus VAS scores (treatment group 1.82 ± 1.15 vs. control group 2.34 ± 1.41, *P* = 0.046) were significantly different between the two groups (Figure 3B; Table 3). After 4 weeks of treatment, the treatment group showed both statistically and clinically significant improvements across all clinical indicators compared with the control group (*P* < 0.05). In detail, the clinical symptom scores reduced to 0.82 ± 0.95 in the treatment group, compared to 1.28 ± 1.07 in the control group; the EASI declined to 0.25 ± 0.29 in the treatment group versus 0.40 ± 0.37 in the control group; the pruritus VAS scores decreased to 0.25 ± 0.56 and 0.64 ± 0.88, and the DLQI dropped to 0.61 ± 1.08 and 1.50 ± 1.83, respectively (Figure 3C; Table 4).

**TABLE 3:** Comparison of clinical indicators between the two groups following 2-week treatment.

Index	Treatment group *(n* = 49)	Control group (*n* = 50)	Difference [95% CI]	*P* value
Clinical symptom score	2.67 ± 1.21	3.10 ± 1.05	−0.43 [-0.88, 0.03]	0.065
EASI	0.91 ± 0.45	1.16 ± 0.67	−0.26 [-0.49, −0.03]	0.028
Pruritus VAS score	1.82 ± 1.15	2.34 ± 1.41	−0.52 [-1.04, −0.01]	0.046
DLQI	3.22 ± 2.92	4.28 ± 3.22	−1.06 [-2.28, 0.17]	0.091

**TABLE 4:** Comparison of clinical indicators between the two groups following 4-week treatment.

Index	Treatment group *(n* = 49)	Control group (*n* = 50)	Difference [95% CI]	*P* value
Clinical symptom score	0.82 ± 0.95	1.28 ± 1.07	−0.46 [-0.87, −0.06]	0.025
EASI	0.25 ± 0.29	0.40 ± 0.37	−0.16 [-0.29, −0.02]	0.020
Pruritus VAS score	0.25 ± 0.56	0.64 ± 0.88	−0.40 [-0.69, −0.10]	0.009
DLQI	0.61 ± 1.08	1.50 ± 1.83	−0.89 [-1.49, −0.29]	0.004

**FIGURE 4:**
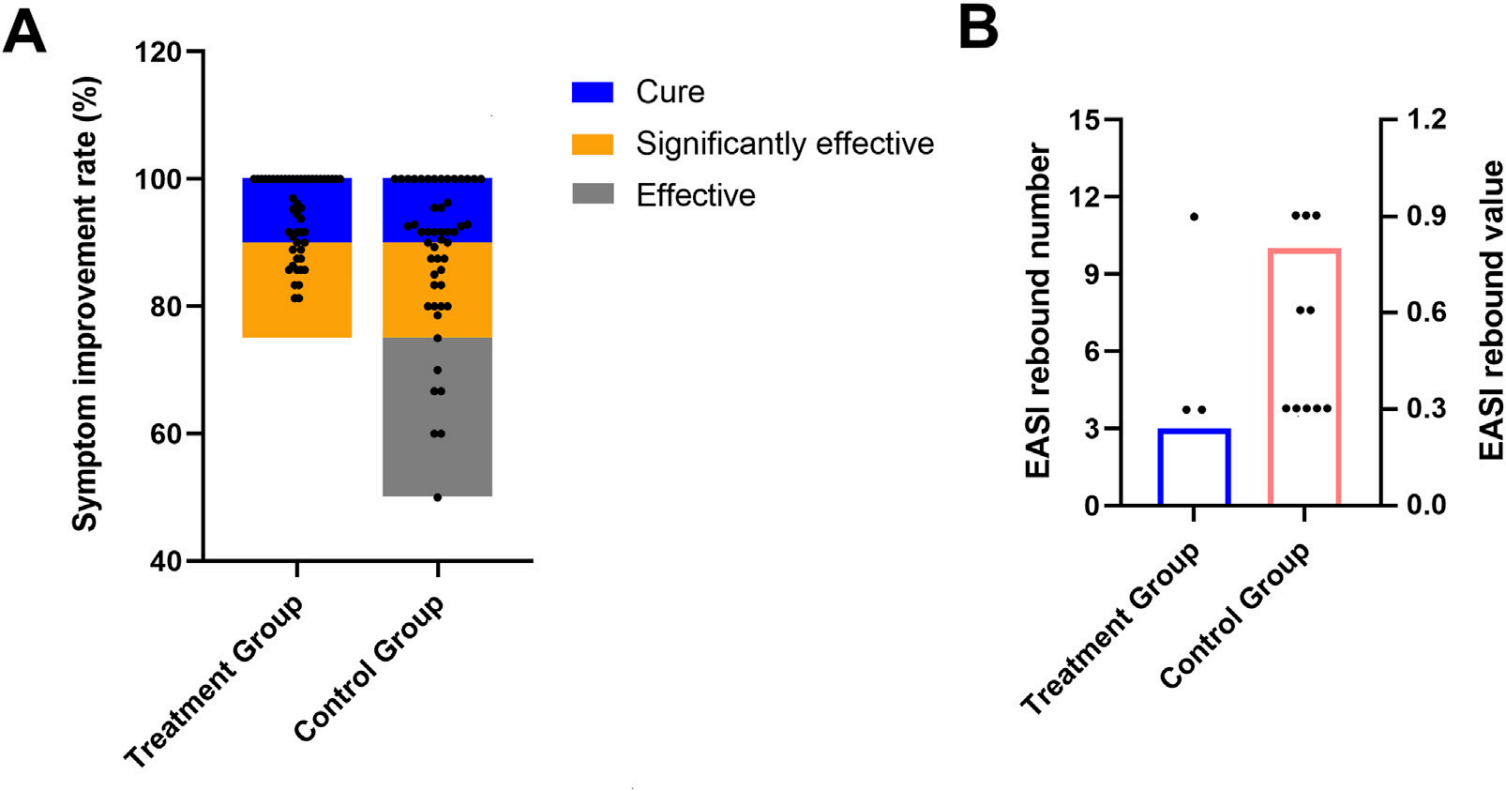
Total significant efficacy of both groups after a 4-week treatment course **(A)**; occurrences of EASI rebound during a 12-week follow-up **(B)**.

Notably, the treatment group yielded a significantly higher “total significant efficacy” compared to the control group (treatment group 100% vs. control group 88%, χ^2^ = 6.573, *P* = 0.037). Specifically, following the 4-week treatment course, 36 out of 49 cases (73.47%) in the treatment group were reported cured, and 13 cases (26.53%) were categorized as significantly effective; while 30 out of 50 cases (60.00%) in the control group achieved cure status, with 14 cases (28.00%) were significantly effective, and 6 cases (12.00%) classified as effective (Figure 4A; Table 5). Collectively, the combination of pimecrolimus cream and APL yields significantly improved efficacy compared to pimecrolimus cream alone in the treatment of CPE.

**TABLE 5:** Comparison of total significant efficacy between the two groups with a 4-week treatment course.

Evaluation of efficacy	Cured (%)	Significantly effective (%)	Effective (%)	Ineffective (%)	*P* value
treatment group (*n* = 49)	73.47	26.53	0.00	0.00	0.037
control group (*n* = 50)	60.00	28.00	12.00	0.00	

### 3.3 Safety evaluation

After the initial application of medications, a few patients reported a mild burning sensation around the anal area, lasting from 2 to 30 min and resolving spontaneously. Generally, no local discomfort was experienced after 1–2 days of treatment. No drug-related serious adverse events were observed in either group, and patients demonstrated good tolerance in the local anal area, with no severe allergic reactions or significant side effects. In detail, 97.96% of patients (48 cases) in the treatment group were classified as Grade I, while 2.04% (1 case) were classified as Grade II. In the control group, 96.00% (48 cases) were classified as Grade I, while 4.00% (2 cases) were classified as Grade II. No patients in either group experienced Grade III or Grade IV adverse reactions (Table 6). Overall, the treatments were safe and well-tolerated, with no severe adverse reactions observed in either group.

**TABLE 6:** Evaluation of drug safety grades in both groups.

Group	Grade Ⅰ	Grade Ⅱ	Grade Ⅲ	Grade Ⅳ	*P* value
treatment group (*n* = 49)	48 (97.96%)	1 (2.04%)	0	0	0.570
control group (*n* = 50)	48 (96.00%)	2 (4.00%)	0	0	

### 3.4 Follow-up survey

After the 4-week treatment, both groups discontinued therapy and were followed up for 3 months (12 weeks). During this follow-up period, clinical symptoms were recorded, and the EASI was recalculated. In the treatment group, 3 out of 49 cases (6.12%) experienced a return or worsening of symptoms, with an EASI rebound, compared to 10 out of 50 cases (20.00%) in the control group (χ^2^ = 4.178, *P* = 0.041) (Figure 4B; Table 7). These findings indicate that pimecrolimus cream combined with APL may potentially lower the relapse rates of CPE, suggesting a possible clinical advantage in maintaining symptom remission.

**TABLE 7:** Comparison of EASI rebound occurrences during a 12-week follow-up between the two groups.

Item	Treatment group	Control group	*P* value
Rebound case (n)	3 (6.12%)	10 (20.00%)	0.041

## 4 Discussion

Chronic perianal eczema (CPE) represents a persistent dermatological condition that is challenging to manage effectively in clinical settings. This study undertook a comprehensive evaluation of clinical indicators and the overall efficacy of pimecrolimus cream combined with a TCM formula (APL) for the treatment of CPE, aiming to provide an innovative clinical treatment regimen.

The integration of Western and traditional Chinese pharmaceutical approaches has been shown to be practical in treating CPE (Al-Ghnaniem et al., 2007). TCM interventions were noted for significantly improving skin integrity, reducing trans-epidermal water loss, normalizing eosinophil levels, and restoring microbial diversity in severe cases of eczema (Maskey et al., 2025). In this study, 99 patients with CPE were categorized into a control group (receiving pimecrolimus cream alone) and a treatment group (receiving both APL and pimecrolimus cream). The changes in clinical indicators and the therapeutic outcomes between the two groups were compared to assess the value of integrating TCM with Western medicine in the treatment of CPE. Both treatments exhibited reductions in clinical symptoms and the EASI scores, relief from itching, and enhancements in skin-related quality of life for patients, indicating favorable therapeutic effects in CPE. Further, the treatment group demonstrated significantly improved outcomes compared to the control group across clinical symptom scores, EASI, pruritus VAS scores, DLQI, and total significant efficacy. Additionally, the relapse rates could potentially be decreased in the treatment group than in the control group. Taken together, the combination of APL with pimecrolimus cream could provide a more effective reduction in CPE symptoms and itchiness, subsequently enhancing patients’ quality of life and lowering the risk of recurrence.

**FIGURE 5:**
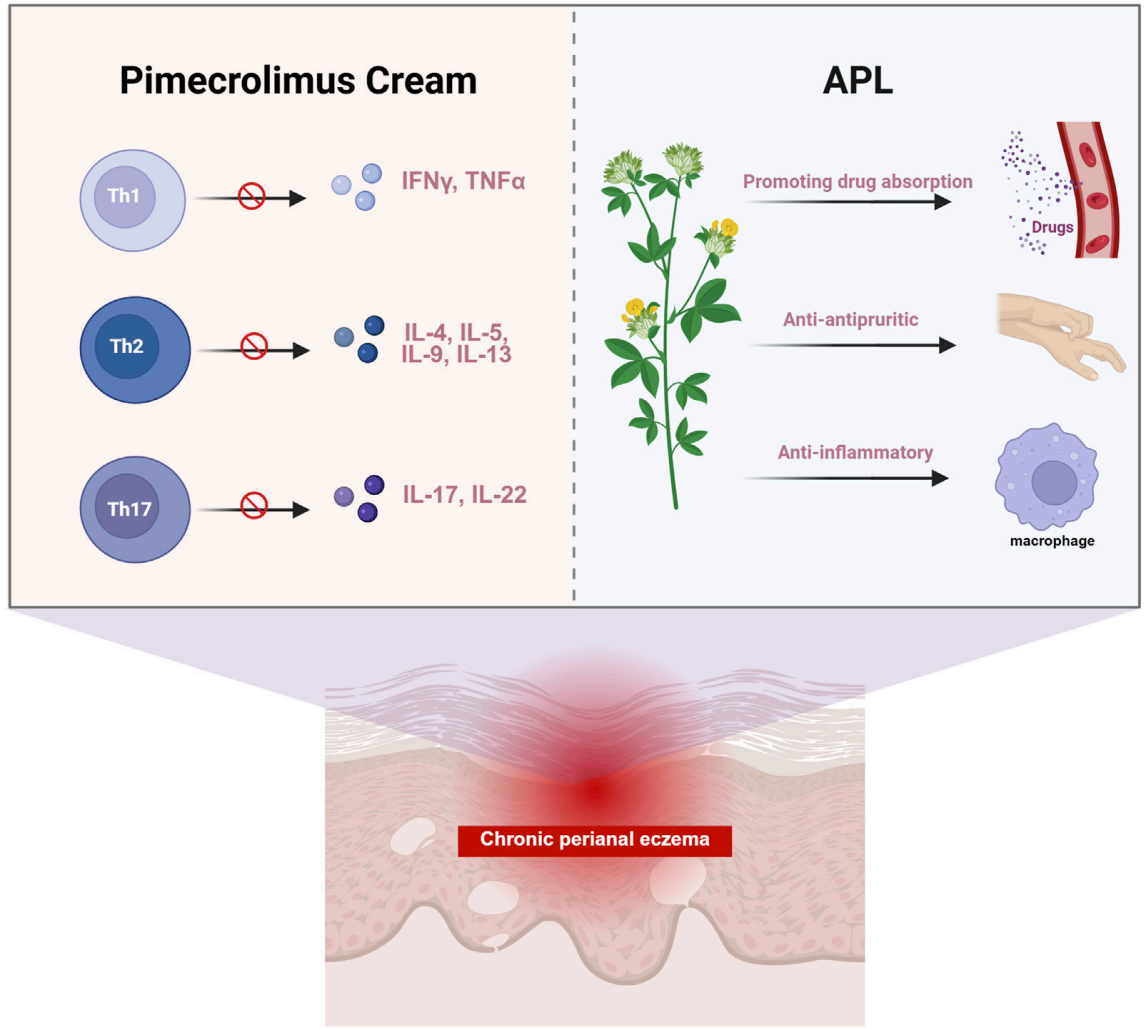
The proposed mechanism for combining APL and pimecrolimus cream in the treatment of CPE.

Pimecrolimus cream is an FDA-approved topical calcineurin inhibitor, with research affirming its effectiveness and safety in controlling eczema (Finlay and Khan, 1994; Gao et al., 2021; Gisondi et al., 2005). Fumigation and sitz-bath therapy, an external treatment of TCM, can facilitate the opening of sweat glands, hair follicles, and sebaceous glands in the skin, which promotes the penetration and the transdermal effects of medications. This treatment approach can also enhance telangiectasia and blood flow and circulation, thus improving metabolism and expediting the repair of skin tissue. In addition, compared to other topical treatment methods, this fumigation and sitz-bath therapy offers the advantages of flexibility in application, personalized treatment options, cost-effectiveness, and high patient acceptance. The APL formula was developed in TCM compatibility theory and clinical practice. Kushen is usually employed in the treatment of eczema dermatitis, exhibiting anti-inflammatory, antipruritic, and immunological functions (Prucha et al., 2013). Huangbo has also become a staple for eczema treatment, as it could decrease mRNA expressions of proinflammatory cytokines (like IL-1β, TNF-α, IL-17, IL-4, and IL-13). Besides this, the mechanism of Huangbo in addressing specific eczema conditions may involve the regulation of T-cell immune balance (Chu et al., 2023). Machixianis has been recognized for its neuroprotective properties, as well as its antibacterial, antioxidant, anti-inflammatory, and anti-ulcer effects (Lin et al., 2023). The clinical application of Jingjie is frequently used in fumigation therapy for eczema dermatitis, while Fuping contains flavonoids and anthocyanins that could help delay skin aging (Zheng et al., 2021; Zhou et al., 2015). Chantui serves specific dermatitis cases, providing anti-inflammatory effects by regulating NLRP3 inflammasome activity (Ren et al., 2021). Fangfeng and Difuzi possess antibacterial, anti-inflammatory, and antipruritic effects, making them popular options for eczema treatment (Liu et al., 2022; Park et al., 2021). Zhiwushe is typically combined with Fangfeng and Danggui for effective eczema management. The synergy of Danggui and Kushen can offer notable anti-inflammatory and antibacterial benefits as well (Zhao et al., 2015). In light of the collective characteristics of these botanical materials, the therapeutic mechanisms of APL are likely multifaceted, encompassing enhanced drug absorption, anti-inflammatory activity, and antipruritic effects (Figure 5).

The current study presents several notable limitations. First, the nature of APL fumigation and sitz-bath interventions precludes blinding in this open-label trail. To mitigate potential performance and assessment bias, each measurement was conducted by two independent certified physicians, and the data analyst was blinded to the experimental design. Next, the follow-up duration was limited to 3 months, leaving long-term recurrence outcomes uncertain. In addition, this study did not include mechanistic or histopathological analyses to elucidate the pharmacological pathways underlying APL’s effects. Future research should aim for larger sample sizes, extended follow-up periods, and integrated laboratory or mechanistic investigations to further validate these findings and uncover the biological mechanisms of the APL formula in the treatment of CPE.

## 5 Conclusion

In summary, combining pimecrolimus cream with traditional APL through fumigation and sitz-bath therapy effectively integrates Western and traditional Chinese medicine in the management of CPE. This approach significantly alleviates pruritus and promotes the healing of skin lesions, confirming the superiority of the combination therapy over pimecrolimus alone. Clinically, this regimen may provide an advanced therapeutic option for CPE by enhancing symptom control and potentially reducing recurrence risk.

## Data Availability

The original contributions presented in the study are included in the article/supplementary material, further inquiries can be directed to the corresponding authors.

## Abbreviations

APL, Anal pruritus lotion; CPE, Chronic perianal eczema; DLQI, dermatology life quality index; EASI, eczema area and severity index; TCM, Traditional Chinese medicine; VAS, visual analogue scale.
